# PARG Mutation Uncovers Critical Structural Determinant for Poly(ADP-Ribose) Hydrolysis and Chromatin Regulation in Embryonic Stem Cells

**DOI:** 10.3390/cells14141049

**Published:** 2025-07-09

**Authors:** Yaroslava Karpova, Sara Piatz, Guillaume Bordet, Alexei V. Tulin

**Affiliations:** Department of Biomedical Sciences, School of Medicine and Health Sciences, University of North Dakota, 501 North Columbia Road, Grand Forks, ND 58202, USA; iaroslava.karpova@und.edu (Y.K.); sara.piatz@ndsu.edu (S.P.); guillaume.bordet@und.edu (G.B.)

**Keywords:** PARG, PARP, poly(ADP-ribosyl)ation, PARylation, stem cells

## Abstract

Poly(ADP-ribosyl)ation is a crucial posttranslational modification that governs gene expression, chromatin remodeling, and cellular homeostasis. This dynamic process is mediated by the opposing activities of poly(ADP-ribose) polymerases (PARPs), which synthesize poly(ADP-ribose) (pADPr), and poly(ADP-ribose) glycohydrolase (PARG), which degrades it. While PARP function has been extensively studied, the structural and mechanistic basis of PARG-mediated pADPr degradation remain incompletely understood. To investigate the role of PARG in pADPr metabolism, we employed CRISPR/Cas9-based genome editing to generate a novel *Parg^29b^* mutant mouse embryonic stem cell (ESC) line carrying a precise deletion within the PARG catalytic domain. This deletion completely abolished pADPr hydrolytic activity, resulting in massive nuclear pADPr accumulation, yet ESC viability, proliferation, and cell cycle progression remained unaffected. Using *Drosophila melanogaster* as a model system, we demonstrated that this mutation completely disrupted the pADPr pathway and halted developmental progression, highlighting the essential role of PARG and pADPr turnover in organismal development. Our results define a critical structural determinant of PARG catalytic function, underscore the distinct requirements for pADPr metabolism in cellular versus developmental contexts, and provide a genetically tractable model for studying the regulation of poly(ADP-ribose) dynamics and therapeutic responses to PARP inhibition in vivo.

## 1. Introduction

**Figure 1:**
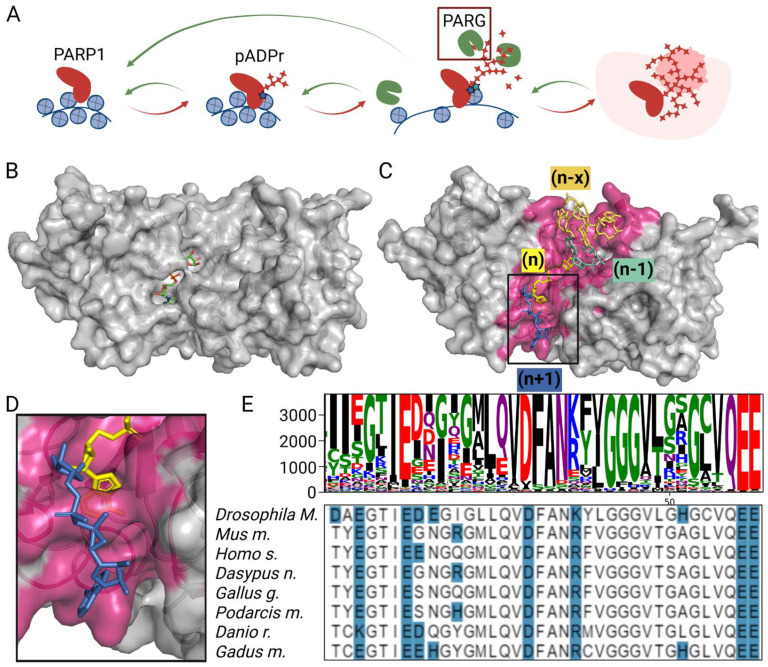
PARG is a catalytic enzyme cleaving poly(ADP-ribose). (**A**) A schematic representation of the poly(ADP-ribosyl)ation pathway regulating the chromatin structure. PARP1 binds to chromatin and becomes activated in response to various stimuli, leading to the synthesis of pADPr (red arrows). This modification primarily occurs through PARP1 automodification, as well as the modification of other chromatin-associated proteins. The accumulation of pADPr causes chromatin relaxation, thereby facilitating transcriptional activation. PARG counterbalances this activity by degrading pADPr chains (green arrows), promoting the dynamic regulation of ADP-ribosylation. Upon intensive automodification, PARP1 dissociates from chromatin and becomes autoinactivated. (**B**) A molecular model of the PARG catalytic domain binding to (n) mADPr. (**C**) A molecular model of the PARG catalytic domain binding to six-ADPr. (n − x) and (n + 1) ADPr positioning predicted by the Chai neuronal network. (n) ADPr is located deep in the PARG catalytic domain pocket. The predicted binding groove for pADPr is highlighted in purple. The magnified binding domain for (n + 1) ADPr unit is presented in (**D**). (**E**) Consensus sequences of the PARG catalytic domain. Protein sequences for selected organisms are presented in the bottom panel (the polar residues are highlighted in blue).

Poly(ADP-ribosyl)ation is a key pathway that coordinates gene expression during development, cell differentiation, and malignancy [1,2,3]. Poly(ADP-ribose) (pADPr) is a long-branched, negatively charged posttranslational modification, which is produced locally in specific chromatin domains. It rapidly loosens the chromatin structure by inducing the phase-separation repulsion of DNA-associated proteins, thereby facilitating chromatin opening and resultant gene expression [3,4,5]. This process is mediated by two groups of enzymes: poly(ADP-ribose) polymerases (PARPs), which modify the target protein with pADPr, and poly(ADP-ribose) glycohydrolases (PARGs), which degrade it (Figure 1A) [1]. In mammals, multiple PARP family members can catalyze poly(ADP-ribosyl)ation, while others function as mono(ADP-ribose) (mADPr) transferases [6]. These enzymes use NAD^+^ as a substrate to add one or more ADP-ribose units, forming polymers that differ in size and branching depending on the specific PARP and cellular context [6,7].

In contrast, while several enzymes are responsible for removing mADPr, only one enzyme, PARG, is capable of effectively degrading poly(ADP-ribose) [6,8]. PARPs have been extensively studied, resulting in significant insights into how they are regulated and activated, as well as the co-factors that influence their binding, targeting, and rate of catalytic activity. In contrast, the role of PARG, which is responsible for pADPr degradation, has only recently gained attention. Studies, particularly in *Drosophila*, have begun to illuminate its function in maintaining balance in the poly(ADP-ribosyl)ation process, highlighting the interplay between PARPs and PARG in regulating cellular homeostasis and tissue functioning [9].

PARG is the only enzyme that can effectively cleave branched and linear poly(ADP-ribose) polymers. Its catalytic domain is built around a conserved macrodomain fold, which is augmented by a PARG-specific extension containing the signature GGG-X_6–8_-QEE motif. This motif is critical in binding ADP-ribose and catalyzing the hydrolysis of the α(1″→2′) O-glycosidic bond via an SN2-like mechanism [10,11,12,13,14]. The catalytic activity of PARG also depends on a conserved “tyrosine clasp” loop, which stabilizes the ribose ring through π–π stacking interactions and helps align the scissile bond [10]. A catalytic glutamate residue is positioned to act as a general acid/base, facilitating nucleophilic attack and bond cleavage [10,11]. While the key amino acids at its active site responsible for hydrolysis have been identified, our understanding of how PARG recognizes and processes the full-length pADPr chain remains limited [11,13]. Current crystal structures are limited to mono- and di-ADP-ribose fragments, providing insufficient insights into the interaction between PARG and longer chain [8,15]. The structural resolution of PARG in a complex with pADPr is hindered by the high conformational flexibility of the polymer, which precludes its capture in crystallographic studies [8]. Meanwhile, computational modeling remains an inherently empirical and approximate approach, often producing results that lack reliability without experimental validation.

In this study, we employed a CRISPR/Cas9-guided approach to generate a *Parg^29b^* mutant embryonic stem cell (ESC) line with a deletion in the loop, responsible for the binding and correct positioning of the (n) ADPr unit and the processing of pADPr, that significantly impairs pADPr hydrolyzing activity. The homozygous *Parg^29b^* mutation led to a massive accumulation of pADPr in the nuclei of ESCs, disrupting the nuclear poly(ADP-ribosyl)ation pathway, which did not affect basic cellular functioning but disrupted organismal development.

## 2. Materials and Methods

**Cells**

ES-E14TG2a cells were purchased from ATCC (Stock # CRL-1821). *Parg^tm2b/wt^Parp1^tm1z/tm1z^* double-mutant ESCs were generated in [16].

**Cell culturing and transfection of ESCs**

Mouse ESCs were routinely cultured in KO DMEM (Gibco, Waltham, MA, USA), 15% KO serum replacement (Gibco), 1x non-essential amino acids (Thermo Fisher, Waltham, MA, USA), 1× Pen/Strep (Thermo Fisher), 1× GlutaMAX (Thermo Fisher), 0.1 mM 2-Mercaptoethanol (Millipore Sigma, St. Louis, MO, USA), 5 µg/mL Insulin, 1 µM PD0325901 MEK inhibitor (Stemcell Technologies, Vancouver, BC, Canada), 3 µM CHIR99021 GSK3 inhibitor (Stemcell Technologies), and 200 U/mL LIF factor (Millipore Sigma) on gelatinized plates [17]. Accutase (Innovative Cell Technologies, San Diego, CA, USA) was used for cell detachment during passaging. To inhibit PARPs, ESCs were cultured in regular media with 7 µM of olaparib (93852, Cell Signaling, Danvers, MA, USA) or rucaparib (S1098, Selleck, Houston, TX, USA). ESCs were transfected in a suspension for 10 min with Lipofectamine 2000 (Invitrogen, Carlsbad, CA, USA), as described [18].

**Molecular cloning and generation of *Parg*-mutated ESCs with CRISPR/Cas9**

The generation of *Parg*-mutated ESCs with CRISPR/Cas9 was performed as described [19]. gRNAs were designed in Benchling software. To generate ESCs with one deleted *Parg* allele, the following gRNAs were used: intron1 gRNA1 (AATAAACTATCAGTCGTCCG) and intron17 gRNA2 (GCATGCGCAGCCGATCCCTG). To target *Parg*, a set of gRNAs against exons was designed and cloned (Table 1). Complementary oligos were annealed, phosphorylated, and cloned into PX458 (#48138, Addgene, Watertown, MA, USA), pDG458 (#100900, Addgene) or pDG461 (#100902, Addgene) plasmids using Golden Gate assembly as described [20]. ESCs were transiently transfected using Lipofectamine 2000 (Invitrogen) with CRISPR/Cas9 plasmids, and 24 h after transfection, GFP-positive ESCs were separated using flow cytometry and seeded in a 10 cm gelatinized dish at 3000 cells per well. Single clones were grown for 7 days, picked up with 10 µL pipette tips, broken into single cells by incubating for 5 min in Accutase (Innovative Cell Technologies), and seeded into separate wells in a 96-well plate. Single clones were further expanded and tested with ELISA, Western blotting, and immunofluorescence staining to assess pADPr levels.

To generate *Parg^29b^* ESC lines with the CRISPR/Cas9 nickase system, the pDG461 (#100902, Addgene) plasmid was cloned by targeting exon 12 of the *Parg* gene with gRNA1 (GAGGGTACCATAGAAGGCAA) and gRNA2 (GTGACGTGTAAGCGTGTCAG). A DNA template for homology recombination was generated by the PCR amplification of the 700bp region in *Parg^29b^* mutant ESCs using the primers FWD (GGTGTTAGTGTGAGTGCATGTGTG) and REV (GCAGGAGGATCACAGGGTTAAGA) and cloned into pEGFP-N3 plasmid. HR homology arms were generated for the transfection of ESCs with HR donor DNA generated from PARG^29b^-pEGFP-N3 plasmid that had been cut with EcoRI and HpaI restriction enzymes (New England Bioscience, Ipswich, MA, USA) and purified with the QIAquick PCR & Gel Cleanup Kit (Qiagen, Germantown, MD, USA). ESCs were transiently transfected with pDG461 plasmid and *Parg^29b^* DNA template, and single clones were generated. Immunofluorescence staining for pADPr was performed to detect successfully targeted clones.

To generate the *Parg* gene tagged with GFP, *Parg^wt^* and *Parg^29b^* ESC lines were transfected with pDG461 (#100902, Addgene) plasmid encoding gRNAs targeting exon 18 of the *Parg* gene with gRNA1 (GACATCCTATTTGAAATGTG) and gRNA2 (TAGGATGTCTCTTTGAGAGG) and DNA template for homology recombination, which was generated from pEGFP-N3-PARG-ex18-GFP plasmid by cutting with HpaI and MfeI and gel extraction. Single ESC clones were generated, and *Parg* tagging in genome was confirmed with Sanger sequencing (Eton Bioscience, San Diego, CA, USA).

To generate PARG expressing plasmids in ESCs, PARG cDNA was amplified from pCMV6-mPARG (SKU MR219292, Origene, Rockville, MD, USA) with PCR using mPARG-cDNA_fwd AAAAGCTAGCGTACCGAGGAGATCTGCCG and mPARG-cDNA_rev AAAAGTCGACGTCAGTGGTCTCTGCACTTGAC primers. PCR fragments and pEGFP-N3 vector were digested with NheI and SalI restriction enzymes (New England Bioscience) and purified with the QIAquick PCR Purification Kit (28104, Qiagen, Venlo, The Netherlands). The treated PCR fragment inserts and vector were ligated with T4 DNA ligase (New England Bioscience M0202), and ligation products were transformed into One Shot Competent *E. coli* cells (C404010, Invitrogen, USA). Colonies were cultured in LB broth (244620, BD Difco, Franklin Lakes, NJ, USA) and extracted with the QIAprep Spin Miniprep Kit (27106, Qiagen, The Netherlands). Catalytically inactive E^748^N/E^749^N PARG^NN^ and deletional ∆TYEG^717^ PARG^29b^ mutant PARG were generated by PCR assembly from pEGFP-N3-mPARG and cloned into empty pEGFP-N3 as described above. Correct cloning was confirmed with Sanger sequencing (Eton Bioscience, USA). The following primers were used: PARGNN_fwd GGTGCGGGACTTGTACAAAATAATATCAGATTTTTAATCAATCCTGAATTGATT, PARGNN_rev CAATTCAGGATTGATTAAAAATCTGATATTATTTTGTACAAGTCCCGCACC, PARG29b_fwd AGCCTCTGACACGCTTACACGTCACCATAGAAGGCAACGGCCGAGGCATG, PARG29b_rev CATGCCTCGGCCGTTGCCTTCTATGGTGACGTGTAAGCGTGTCAGAGGCT. 

To generate pUAST-mPARG-GFP plasmids with PARG^wt^, PARG^NN^ or PARG^29b^ variants, the gene was cloned from pEGFP-N3-mPARG plasmid into the pUAST vector with BglII and NotI restriction enzymes (New England Bioscience).

The DNA template for homology recombination to tag the *Parg* gene in ESCs with GFP with CRISPR/Cas9 was generated from three pieces. The left arm was amplified via PCR from the genome with the primers LA_F AAGCTTCGAATTCTGCAGTTGGACAGAGCATAGTAAGAAATG and LA_R CGCGGTACCGTCGACGTCAGTGGTCTCTGCACTT, GFP was amplified from pEGFP_N3 plasmid with GFP_F GCAGAGACCACTGACGTCGACGGTACCGCGG and GFP_R TGCCTTCTGTCCTGGTTACTTGTACAGCTCGTCCAT, and the right arm was obtained as a gBlock (IDT, San Diego, CA, USA) with mutated gRNA sites: CCAGGACAGAAGGCAGGCACCTGAGGAACAAGTGACTAGAAGCTCCTCTCAAAGAGACATCCTATTTGAAATGTGAAGTGTGATGTCTGAATTGACTGAATCTGATCTAAGTGTGTATATAATCCACATTTGTAATCAAGGATGCAGTCTCTTCTGCATATGCAGTTGTTTCTTGTTCATCCTGGTGGACATGCCTTTAGACATGGCTTCTTCAATTTTTCTTCTCCTTCAGTCTTTATTCTTTGATTTTTTTTTTCCAACTTGATTTCTTGGGAAAACTCAAGAAAGGTTGCACTCAGCTTCTAGATCTTTCTCTTCCTGTCTGTGTGTTGTCCAGACTGCTTTGGTGGCTAGCAGATACCATCACACTTGGAGGAAGTTACAAATCCAGAAATCTGAGTTTGCTGCAGATTTACCTGTGAGCTTCTCACTCCCAACCCTTGTTAGGCTTGTGTTGTCTACATTTTCAATTTTGGAAGTTGAAGTTTTTCTTATGTTACTTAATGCTAGTATCTTTTAGGCTAAAACTATTTTCTATTTAAGGCAGACTAATTTCCAGTTTCTCTTTTGAAACATCATCCCTATAAGTAACGGTTTTTTTCGTCCTTTTTTCCCCAGCGCTATTTTAGAAGCTGGCCAAGAGGAAAGAAAATGTAGAATAAAAGGATTTTCCTCGGATGCTATAAAGAAGCCAGGTTCAAGAGCGTTGGGGTTTTTGTTTTTTTCAAGACTTGTTTTTCCTTTGCAGCTAGGGTGAGTGCTTGTTCTGTGGTGCTGAGGGCATAGTCCTGTAACCAAAGGTCT.

Three DNA parts were assembled via PCR using the primers LA_NotI_F9.

TTTGCGGCCGCTTTATTGATACAGAAATCTACAGGGG RA_MfeI_R3 AAACAATTGAGACCTTTGGTTACAGGACTATG and cloned into pEGFP_N3 plasmid with NotI (New England Bioscience) and MfeI (New England Bioscience) to store as pEGFP-N3-PARG-ex18-GFP.

**ELISA**

Maxisorp plates were coated with 0.25 µg/mL pADPr H10 antibodies (sc-56198, Santa Cruz, CA, USA) overnight, blocked with 5% nonfat milk (Rockland, ME, USA), and incubated with lysed cells overnight at 4 °C. After plates were washed, pADPr-antibody conjugates were stained with pADPr reagent (1:1500, MABE1031, Millipore Sigma) and secondary anti-rabbit antibodies (1:1000, Perkin Elmer, Waltham, MA, USA). Reaction was developed with SureBlue reagent (Seracare Life Sciences Inc., Milford, MA, USA), and absorbance was measured using a Cytation 3 Multi-Mode Reader (BioTek, Shoreline, WA, USA).

**Western blotting**

Western blotting was performed as described previously [2]. Briefly, ESCs or Drosophila third instar larvae were lysed in RIPA buffer (Thermo Fisher), mixed with 2 × sample loading buffers (Bio-Rad), and heated at 99 °C for 5 min. Proteins were separated in 14% Polyacrylamide gel (Thermo Fisher) and transferred to a nitrocellulose membrane. Membranes were blocked with 5% milk (Rockland) for 1 h and stained overnight at 4 °C with primary antibodies against pADPr H10 (1:1000, sc-56198, Santa Cruz), β-actin (1:5000, A2228, Millipore Sigma), H3 (1:3000, ab1791, Abcam, Cambridge, MA, USA), PARP1 (1:1000, ab6079, Abcam), or a special reagent against pADPr (1:2000, MABE1031, Millipore Sigma), along with the corresponding secondary antibodies, anti-mouse HRP (1:3000, G21040, Invitrogen) and anti-rabbit HRP (1:1500, Perkin Elmer), for 1 h at room temperature.

**Immunofluorescence staining**

ESCs were fixed in 4% PFA (Thermo Fisher) for 15/30 min at room temperature, permeabilized with 0.3% Triton-X100 (Millipore Sigma) for 30 min, blocked in 5% normal goat serum (Abcam) and 0.1% Triton-X100 (Millipore Sigma) on PBS (Millipore Sigma), and stained with antibodies diluted in blocking solution. Primary antibodies for overnight staining at 4 °C were used against pADPr (1:500, sc-56198, Santa Cruz), GFP (1:1000, ab290, Abcam), and a special reagent against pADPr (1:2000, MABE1031, Millipore Sigma). The corresponding secondary antibodies used were anti-mouse Alexa 488 (1:1000, A28175, Invitrogen) and anti-rabbit Alexa 568 (1:1000, A-11011, Invitrogen) for 45 min at RT. Draq5 (Thermo Fisher) or TOTO3 (Biotium, Fremont, CA, USA) was used as a DNA marker. Confocal imaging was performed using a Leica DMi8 microscope.

**Analysis of confocal images**

To segment single-cell nuclei in confocal images, Napari software was used with the StarDist deep-learning neural network plugin trained to detect fluorescent-labeled nuclei [21,22]. Nuclei segmentation was performed in the DNA Draq5 or TOTO3 channel, and the pADPr signal intensity was measured in the corresponding channel.

**Flow cytometry analysis**

ESCs in a single-cell suspension were fixed in 2% paraformaldehyde (Thermo Fisher) for 15 min at room temperature, permeabilized, and blocked in 5% normal goat serum (Abcam), 1% bovine serum albumin (Millipore Sigma), and 0.7% Tween 20 (Millipore Sigma) on PBS for 15 min at room temperature and then stained with primary antibodies diluted in blocking solution overnight at 4 °C and with secondary antibodies for 30 min at room temperature. The primary antibodies used were pADPr (1:500, sc-56198, Santa Cruz), phSer10H3 (1:1000, 9701, Cell Signaling), BrdU (1:500, 5292S, Cell Signaling), and a special reagent against pADPr (1:2000, MABE1031, Millipore Sigma). The corresponding secondary antibodies used were anti-mouse Alexa 488 (1:1000, A28175, Invitrogen) and anti-rabbit Alexa 568 (1:1000, A-11011, Invitrogen). For cell cycle analysis, cells were fixed with ice-cold 70% ethanol for 30 min on ice and stained with FxCycle™ PI/RNase Staining Solution (Thermo Fisher). For the BrdU incorporation assay, cells were pretreated with BrdU (Thermo Fisher) for 35 min. Single cells were then fixed in 70% ethanol, treated with 1 N HCl (Millipore Sigma) for 30 min, and stained with FxCycle™ PI/RNase Staining Solution (Thermo Fisher). Flow cytometry for all experiments was performed on a BD FACSymphony™A3 Cell Analyzer (BD Bioscience, Franklin Lakes, NJ, USA), and analysis was performed using FlowJo software (version 10.9).

**Protein modeling**

Models of deletional mutants in the PARG catalytic domain were generated with RosettaFold, AlphaFold, or Chai-1 [23,24]. PARG catalytic domain modeling with different pADPr lengths was performed using a foundation model for molecular structure prediction, Chai-1. The PARG catalytic domain structure (PDB ID:4FC2) was used as a template for aligns [25]. All protein structures were analyzed using PyMOL v3.1.0.

**Transgenic *Drosophila melanogaster* line generation and crossing.**

Flies were reared at 20 °C unless otherwise stated. The transgenic stock with P{w1, UASt::PARG-GFP} were generated by the injection of pUAST-mPARG-GFP plasmids into embryos. The *parg^27.1^* mutant, PARP1:DsRed stock, and *69B-GAL4* driver were generated elsewhere [26].

**Statistics**

Statistical analyses were performed using a 2-tailed Student’s *t*-test. A *p*-value of 0.05 or less was considered significant. The results were analyzed using the indicated statistical test in GraphPad Prism (9.4.0).

## 3. Results

### 3.1. Critical Loop in PARG Catalytic Domain Regulates Poly(ADP-Ribose) Processing

Although the positioning, binding, and cleavage of di(ADP-ribose) by PARG are well defined, the mechanisms underlying PARG’s interaction with extended pADPr chains and its ability to perform suggested processive cleavage remain unclear [11,12]. Structural studies have been limited by the polymer’s intrinsic flexibility—only the terminal dimer has been resolved from 6- to 16-mer oligomers, with additional (n − x) ADPr units positioned distally from the catalytic site [8]. Similarly, the modeling of human and mouse PARG bound to five-unit oligomers by a trained AI model consistently placed the folded oligo(ADP-ribose) (oADPr) in a distal pocket away from the (n) ADPr unit (Figure 1B,C and Figure S1). However, crystallographic studies across species consistently show that the terminal (n) ADPr binds tightly within the catalytic domain, whereas the (n − 1) ADPr is only weakly associated, with its ribose moiety lacking direct contact with any amino acid residues [8,15,27]. Moreover, the adenine of the terminal (n) unit is essential in diADPr derivative cleavage [15]. These findings conflict with models proposing that PARG acts in a processive manner by remaining anchored at the (n) terminus and releasing successive (n − 1) fragments along the pADPr chain (Figure 1C) [25]. We hypothesized that residues adjacent to the (n + 1) ADPr binding region might contribute to pADPr chain interaction (Figure 1D). They form two loops, which are present across different species and possess a set of conservative amino acids (Figure 1D,E).

**Figure 2:**
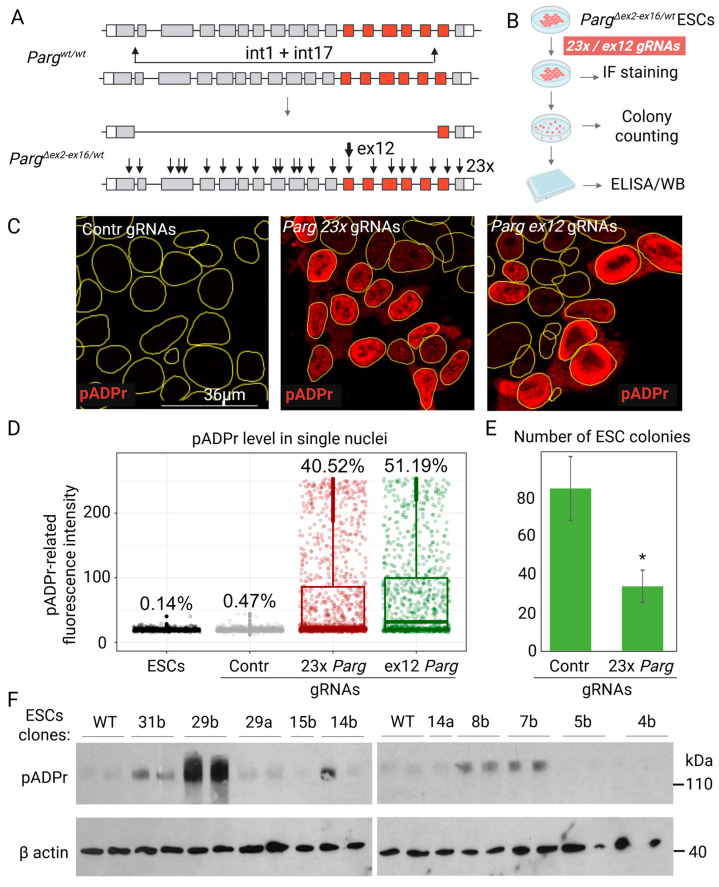
Generation of *Parg* mutant ESCs. (**A**) Schematic representation of *Parg* gene alleles generation in ESCs. Introns 1 and 17 of the wild-type *Parg* allele was targeted with the CRISPR/Cas9 system, and the *Parg^∆ex2-ex16/wt^* ESC clonal line was generated with one allele deleted. *Parg^∆ex2-ex16/wt^* ESCs were targeted with gRNAs against either 23 sites at exons or exon 12 of the remaining *Parg* allele. The exons encoding the catalytic domain are highlighted in red. (**B**) Targeted ESCs from (**A**) were split for three experiments. The pADPr level in single nuclei was detected in a mixed population 48 h after transfection using immunofluorescence (IF) staining. Single clones were generated and counted. Single clones of ESCs were established and screened to assess their pADPr level using ELISA and Western blot. (**C**) Fluorescence staining of single nuclei of mixed ESCs 48 h post transfection with the CRISPR/Cas9 system targeting *Parg* gene exons or control with pADPr H10 antibody. The accumulation of pADPr in the nuclei of targeted cells is shown. Single nuclei are highlighted with yellow lines. (**D**) The quantification of pADPr levels in single nuclei from (**C**). The percentage of nuclei with a pADPr-related fluorescence level higher than 30 are shown on the plot for each condition. (**E**) *Parg^∆ex2-ex16/wt^* ESCs were targeted toward 23 sites in *Parg* gene exons or with random gRNAs. *Parg*-targeted ESCs formed significantly fewer colonies. Data are presented as the mean ± SD (*n* = 3; ∗ *p* < 0.01 by Student’s *t*-test). (**F**) Selected Western blotting to check ESC clones after targeting the *Parg* gene with CRISPR/Cas9 against exon 12. Samples were stained with antibodies against pADPr H10 and β actin for loading control.

To study the function of this region in cells, we used the CRISPR/Cas9 system to target it in mouse embryonic stem cells (ESCs), a system well suited to clonal screening and possessing high PARP1 levels. To increase the targeting efficiency, we engineered *Parg^ex2-ex16/wt^* ESC lines with one *Parg* allele missing exons 2–16 by directing Cas9 nuclease to corresponding introns with two gRNAs (Figure 2A). Single clones of *Parg^ex2-ex16/wt^* ESCs were isolated and confirmed by PCR to have retained only one intact *Parg* allele (Figure 2A).

Previous reports suggest that complete *Parg* deletion is incompatible with the maintenance of cells in vitro with intact PARP1 [28]. To confirm this, we transfected generated *Parg^ex2-ex16/wt^* ESCs with CRISPR/Cas9 plasmids targeting 23 distinct regions across *Parg* exons (Figure 2A,B). Immunofluorescence in pADPr 48 h post transfection showed that around 40% of cells accumulated high levels of pADPr, verifying gRNA activity (Figure 2C,D). However, the ELISA screening of >250 single colonies revealed no clones with similar pADPr accumulation, and colony formation was markedly reduced when cells were plated at a low density post transfection, confirming that complete *Parg* loss prevented ESC growth in vitro (Figure 2E).

We next employed a single gRNA targeting the region near the (n + 1) ADPr unit, which forms a loop at the entrance of the PARG catalytic domain (Figure 1D,E). As frameshift mutations would result in complete loss of PARG, a condition shown above to be incompatible with cell maintenance, only small in-frame deletions were expected to be recoverable. Exon 12 of *Parg* was targeted in *Parg^ex2-ex16/wt^* ECSs, and single-cell clones were expanded and screened for pADPr accumulation by ELISA, followed by the Western blot analysis of selected clones (Figure 2B,F). Among the generated clones, clone #29b exhibited a more than tenfold increase in pADPr levels compared to control cells, indicating severely impaired PARG catalytic activity (Figure 2F).

### 3.2. The Novel Mutation in Parg Locus Completely Impairs pADPr Catabolism in ESCs

**Figure 3:**
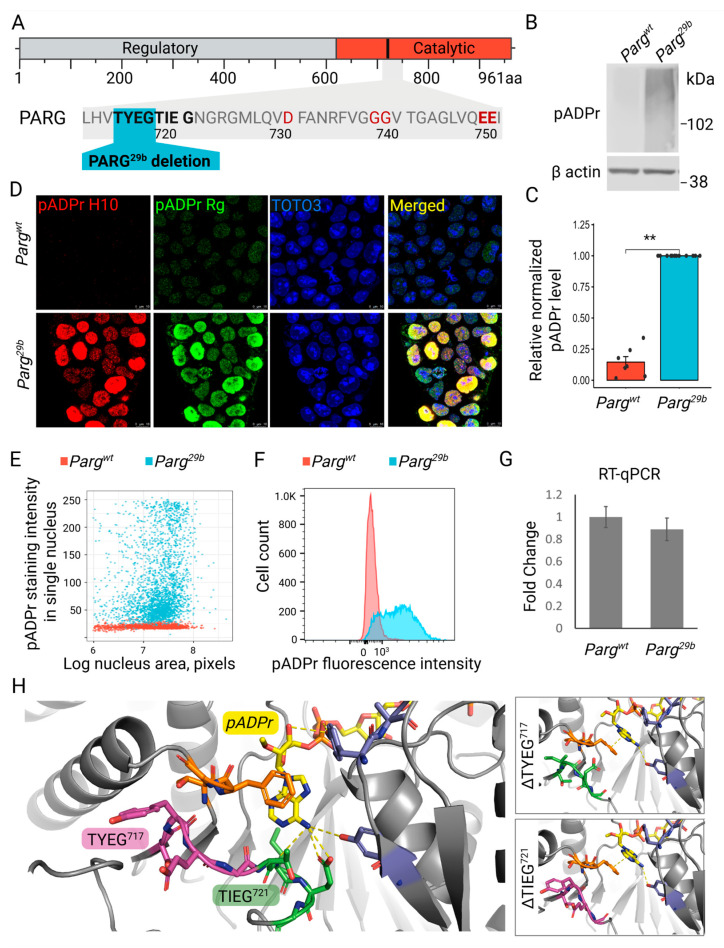
The *Parg^29b^* mutation leads to pADPr degradation deficiency in ESCs. (**A**) A schematic representation of the PARG protein with a 29b mutation generated by CRISPR/Cas9. The key catalytic residues are highlighted in red. (**B**) Western blotting analysis showing the accumulation of pADPr in *Parg^29b^* ESCs. β actin is shown as a loading control. (**C**) The quantification of Western blotting from (**B**). Each dot represents one experiment, and the level of pADPr is normalized to *Parg^29b^* ESCs (∗∗ *p* < 0.01 by Student’s *t*-test). (**D**) Representative images of stained ESCs for pADPr with antibodies (pADPr H10) or special reagent (pADPr Rg), demonstrating the accumulation of pADPr in *Parg^29b^*-mutated ESCs. DNA stained with TOTO3. (**E**) The fluorescence intensity of pADPr H10 antibody staining in single nuclei of ESCs from (**D**). (**F**) Flow cytometry of ESCs stained with antibodies against pADPr H10. (**G**) RT-qPCR for *Parg* mRNA level in control and *Parg^29b^* ESCs showing no statistically significant difference. (**H**) The structural model of PARG catalytic domain binding (n) ADPr. Predictions of structural changes for TYEG^717^ and TIEG^721^ mutations are shown in the right panels.

Sanger sequencing identified a ∆TYEG^717^ deletion in the mutant #29b allele of *Parg* (Figure 3A). This deletion led to robust pADPr accumulation, confirmed by Western blotting, immunofluorescence, and flow cytometry using both antibody- and macrodomain-based detection (Figure 3B–F). The reduction in pADPr levels after cells were treated with PARPs inhibitors confirmed its nature and the specificity of detection (Figure S2). Total *Parg* mRNA levels in this clone were comparable to the wild-type, confirming that the phenotype was not due to transcriptional downregulation (Figure 3G). Because no commercial antibodies are available to detect mouse PARG, we generated wild-type and *Parg^29b^* ESC lines in which the *Parg* gene was tagged with GFP in the genome. Western blot analysis demonstrated comparable levels of PARG:GFP in both cell lines, and no degradation of the PARG^29b^ protein was observed (Figure S3). These results support the conclusion that the mutation does not affect PARG expression or protein stability. To confirm that pADPr accumulation is specifically caused by the PARG mutation rather than off-target effects, we generated ESCs harboring the ∆TYEG^717^ mutation using a CRISPR/Cas9 nickase-based knock-in approach. All independently derived clones exhibited similarly elevated pADPr levels, validating the causative role of the mutation (Figure S4).

To understand what structural changes disrupt the PARG^29b^ enzyme’s ability to efficiently cleave pADPr, we predicted its structure with different trained AI models. Surprisingly, all predictions replaced the deleted TYEG^717^ loop with the following loop, TIEG^721^, keeping the preceding β sheet intact (Figure 3H and Figure S5). The crystal structure of the mouse PARG catalytic domain demonstrates a direct bond between E and the adenine from terminal ADPr. The removal of either TYEG^717^ or TIEG^721^ loops is predicted to affect not only the binding of this adenine but also its position relative to phenylalanine F^895^ (Figure 3H). The aromatic rings of adenine and F^895^ create a π-stack bond that is proposed to play an important role in the proper positioning of pADPr and the interplay with tyrosine clasp in the active center of PARG and its release upon the completion of cleavage [10].

**Figure 4:**
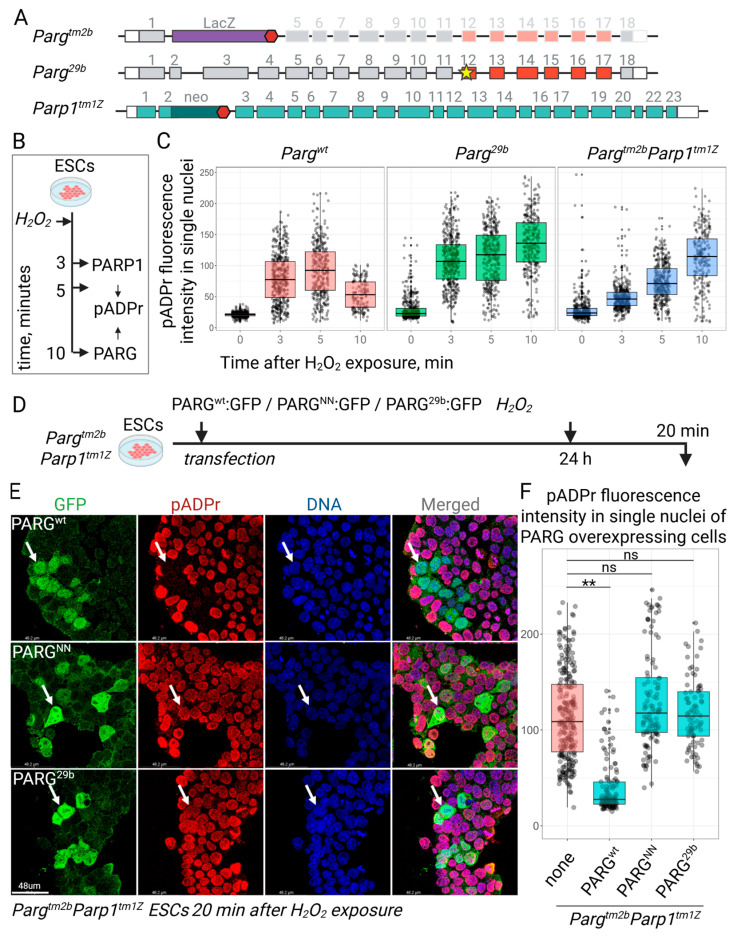
PARG^29b^ unable to degrade pADPr. (**A**) *Parg* and *Parp1* mutant alleles used in the current study. *Parg^tm2b^* is a total *Parg* knockout, *Parg^29b^* is the allele with deleted nucleobases corresponding to the TYEG^717^ loop generated in the current study, *Parp1^tm1Z^* is a incomplete *Parp1* knockout with reduced, but still high, poly(ADP-ribosyl)ation activity. (**B**) Mutant ES lines were treated with hydrogen peroxide and then stained for pADPr levels using immunofluorescence at defined time points post exposure. (**C**) Fluorescence intensity reflecting pADPr levels was measured in individual nuclei segmented based on DNA staining after H_2_O_2_ exposure. In control cells with functional PARG, pADPr levels increased at 3 and 5 min after exposure and had significantly declined by 10 min. However, in PARG-deficient ES cell lines, pADPr levels did not decrease at any observed time point. (**D**) PARG-deficient *Parg^tm2b/tm2b^Parp1^tm1Z/tm1Z^* ESC lines were transfected with control PARG^wt^, catalytically inactive PARG^NN^, or PARG^29b^, each tagged with GFP. pADPr accumulation was triggered by hydrogen peroxide treatment, and cells were stained for pADPr and DNA using immunofluorescence 20 min after exposure. (**E**) Fluorescence intensity reflecting pADPr levels was measured in individual nuclei segmented based on DNA staining after H_2_O_2_ exposure, as described in (**D**). In untransfected cells, where intrinsic PARG was absent, the pADPr level remained elevated at this time point. Cells expressing the normal PARG^wt^ variant showed a significant reduction in pADPr levels. However, neither the catalytically inactive PARG^NN^ nor PARG^29b^ was capable of cleaving pADPr and reducing pADPr levels. Selected transfected cells expressing different PARG variants are indicated by white arrows. (**F**) The quantification of pADPr levels in single nuclei from (**E**) (∗∗ *p* < 0.01, ns, not significant).

To further assess the pADPr-degrading efficiency of the PARG^29b^ mutant enzyme, we generated an ESC line with the complete knockout of *Parg*. The inability of ESCs to be sustained in vitro without PARG was circumvented by introducing a hypomorphic mutation into *Parp1* (Figure 4A) [16]. To generate double-mutant *Parg^tm2b/tm2b^Parp1^tm1Z/tm1Z^* ESCs, we crossed heterozygous *Parg^tm2b/wt^Parp1^tm1z/tm1z^* mice with total knockout *Parg* and hypomorph *Parp1* alleles and established independent ESC lines from blastocysts (Figure 4A). Although the homozygous mutation of both enzymes simultaneously is embryonically lethal, we successfully established the double-mutant ESC lines. To evaluate pADPr hydrolysis efficiencies in the ESC lines studied, we induced robust pADPr production by exposing cells to H_2_O_2_ and monitored the clearance of excessive pADPr over time in individual nuclei using immunofluorescence (Figure 4B). In control cells, the pADPr level peaked at 5 min post induction and had decreased significantly by 10 min post exposure (Figure 4C). However, in both *Parg^29b^* and *Parg^tm2b/tm2b^Parp1^tm1Z/tm1Z^* cells, pADPr levels continued to rise at 10 min post exposure, indicating a failure to degrade pADPr in the absence of PARG, or with the TYEG^717^ mutation in, PARG (Figure 4C). Furthermore, the transfection of *Parg^tm2b/tm2b^Parp1^tm1Z/tm1Z^* ESCs with plasmids encoding wild-type PARG^wt^, PARG^29b^, or catalytically inactive PARG^NN^ (E^748^N/E^749^N) revealed that only wild-type PARG was able to restore pADPr levels to the baseline by 20 min post H_2_O_2_ treatment (Figure 4D–F). Cells transfected with mutant PARG variants retained high nuclear pADPr levels, similarly to untransfected knockout cells (Figure 4E,F). These results provide strong evidence that the deletion of the TYEG^717^ loop in PARG abolishes its ability to cleave pADPr.

### 3.3. ESC Proliferation and Survival Are Maintained in the Absence of Functional Parg

**Figure 5:**
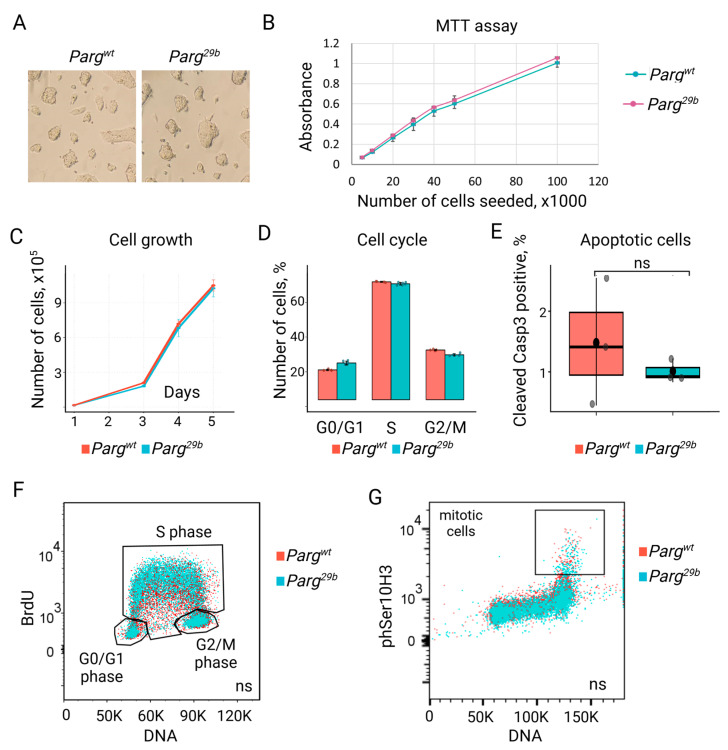
Mutating *Parg* does not affect basic cellular functions. (**A**) Similar morphology of control and *Parg^29b^* ESC colonies. (**B**) MTT assay for control and mutant *Parg^29b^* ESCs, showing no statistically significant difference in cell viability between these two cell lines. Cells were seeded at indicated numbers and grown for 3 days. (**C**) The growth of ESCs is not affected by *Parg^29b^* mutation. (**D**) Cell cycle analysis of *Parg^29b^* ESCs compared to control cells. Data are presented as the mean ± SD. (**E**) Box plots showing similar apoptotic levels in *Parg^29b^* and control ESCs. Cells were stained with cleaved Casp3 antibodies and analyzed by flow cytometry (ns, not significant). (**F**) Flow cytometry analysis demonstrates the same level of BrdU accumulation in *Parg*-mutated and control ESCs. ESCs were treated with BrdU for 30min, fixed, and stained with anti-BrdU antibodies and propidium iodide for DNA. (**G**) Flow cytometry analysis demonstrates a similar percentage of cells undergoing mitosis in *Parg^29b^* and control ESCs. Cells were stained with mitotic marker H3phSer10 antibodies and propidium iodide for DNA.

During routine culture, *Parg^29b^* ESCs displayed no observable differences in growth rate or morphology compared to control ESCs (Figure 5A). Growth curve analysis confirmed the absence of significant changes in proliferation dynamics (Figure 5B). Nevertheless, identical doubling times could still mask opposing effects on cell cycle progression or cell death. To assess these possibilities, we compared cell cycle profiles and apoptosis levels between control and mutant *Parg^29b^* ESCs (Figure 5B–G). Flow cytometric analysis in cleaved caspase-3 revealed no significant differences in apoptosis between groups (Figure 5E). Similarly, cell viability assessed via an MTT assay and S phase entry measured by BrdU incorporation were comparable between mutant and control ESCs (Figure 5B,F). DNA content analysis using propidium iodide staining, alongside mitotic marker phospho-histone H3 (Ser10), showed no alterations in the distribution of cells across G0/G1, S, G2, or M phases (Figure 5D,F,G). PARP enzymes utilize NAD^+^ as a substrate to synthesize poly(ADP-ribose) pADPr. To determine whether the disruption of PARG and the resulting accumulation of pADPr lead to the depletion of the cellular NAD^+^ pool, we assessed the pADPr synthesis capacity under oxidative stress. *Parg^29b^* ESCs were treated with hydrogen peroxide, and pADPr levels were evaluated by Western blotting. The results showed that *Parg^29b^* ESCs were able to further elevate pADPr levels beyond those observed under basal conditions (Figure S6), indicating that these cells retain an excessive pool of free NAD^+^ and that NAD^+^ availability is not limiting under these conditions. Together, these findings indicate that the *Parg^29b^* mutation does not impair the core cellular processes required for ESC proliferation and viability under standard conditions.

### 3.4. PARG^29b^ Mutation Abolishes pADPr Hydrolysis In Vivo and Developmental Progression

**Figure 6:**
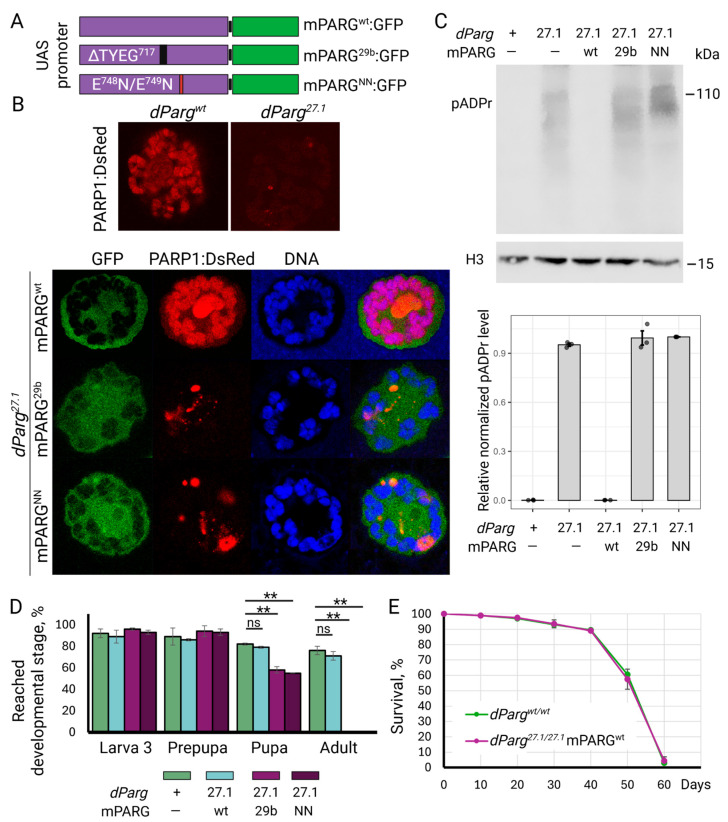
PARG^29b^ mutation unable to degrade pADPr in vivo and support the development of *Drosophila*. (**A**) Schematic representation of genetic constructs used to generate transgenic *Drosophila melanogaster* stocks. Three variants of mouse PARG coding sequence were introduced under the control of UAS promoter: the wild-type (PARG^wt^), the 29b mutant (PARG^29b^), and a catalytically inactive E^748^N/E^749^N (PARG^NN^), tagged with GFP. (**B**) Confocal imaging of single nuclei of salivary gland cells expressing PARP1 tagged with DsRed on the wild-type or on the *Parg^27.1^* knockout background, shown in the top panel. Confocal imaging of single nuclei of salivary gland cells expressing different versions of mouse PARG from (**A**) tagged with GFP (green) and PARP1 tagged with DsRed (red) on a *Drosophila Parg^27.1^* knockout background, shown in the bottom panel. DNA is stained with Draq5 (blue). The restored normal localization of PARP1 is shown only for the wild-type PARG enzyme. (**C**) Western blotting of crude extracts from Drosophila third instar larvae stained with antibodies against pADPr and histone H3 for loading control. The quantification of Western blotting is shown in the bottom panel. Each dot represents one experiment, and the level of pADPr is normalized to the PARG^NN^ version. (**D**) The proportion of flies reached different developmental stages for different transgenic *Drosophila* lines. Only wild-type flies and flies carrying the mouse PARG^wt^ on a *Parg*^27.1^ knockout background were able to complete their development. Results are based on three biological replicates; the SD is shown as error bars (∗∗ *p* < 0.01, ns, not significant). (**E**) Survival plots illustrate the lifespan of adult wild-type flies or flies carrying mouse PARG^wt^ on a *Parg*^27.1^ knockout background. The vertical axis denotes the proportion of surviving individuals; the horizontal axis shows the days following adult emergence. Results are based on three biological replicates; the SD is shown as error bars.

The total knockout of *Parg* in *Drosophila melanogaster* leads to a severe accumulation of pADPr, the mislocalization of PARP1 from chromatin, and developmental arrest during early metamorphosis [29]. We demonstrated that the expression of mouse wild-type PARG fully rescues this phenotype, restoring pADPr turnover, proper PARP1 localization, and viability (Figure 6). This functional substitution underscores the evolutionary conservation of PARG activity and establishes *Drosophila* as a robust in vivo model to evaluate the catalytic competence and developmental consequences of the PARG^29b^ mutation.

We generated three transgenic fly lines expressing GFP-tagged mouse PARG variants under the control of UAS promoter: wild-type PARG (PARG^wt^), the 29b mutant (PARG^29b^), and a catalytically inactive mutant (PARG^NN^; E^748^N/E^749^N) (Figure 6A). All three constructs exhibited similar levels of expression and cellular distribution: a low cytoplasmic content, an enrichment in nucleoplasm, and a distinct chromatin-associated pattern (Figure 6B and Figure S7). No stability issues were detected for mutant versions of PARG, as all forms appeared as a single band in Western blot analysis (Figure S7). To test the functional relevance of the PARG^29b^ mutation, we introduced the transgenes into a *Drosophila* background *Parg^27.1^* lacking endogenous PARG that possessed a transgene expressing PARP1 protein tagged with DsRed. As expected, PARG-null *Parg^27.1^* flies accumulated high levels of pADPr and exhibited the complete delocalization of PARP1 from chromatin, and arrested development at the onset of metamorphosis (Figure 6B–E). The expression of PARG^wt^ fully rescued this phenotype: pADPr levels normalized, PARP1 localized correctly to chromatin and nucleoli, and flies developed into viable, fertile adults (Figure 6B–E). In contrast, neither PARG^29b^ nor PARG^NN^ restored normal function when expressed on a *Parg^27.1^* knockout background. Both transgenes failed to reduce pADPr accumulation or correct PARP1 mislocalization, which occurred due to a lack of endogenous *Drosophila* PARG in *Parg^27.1^* flies, and the developmental arrest persisted (Figure 6B–E). These findings confirm that the PARG^29b^ mutation, like the catalytically inactive PARG^NN^, abolishes the enzymatic hydrolysis of pADPr and disrupts organismal development despite unaltered subcellular localization, but does not strongly impact basic cellular functioning. The data underscore the essential role of pADPr turnover in chromatin regulation and developmental progression.

## 4. Discussion

This study identifies a critical structural motif within the catalytic domain of PARG that is essential for the hydrolysis of poly(ADP-ribose) (pADPr). While the catalytic core of PARG and its interaction with short ADPr units have been well characterized, the mechanism by which PARG accommodates and degrades extended pADPr chains remains poorly understood. Our results demonstrate that deletions within the (n) and (n + 1) ADPr interaction domain completely abolish PARG activity, leading to a pronounced nuclear accumulation of pADPr without significantly affecting the overall cell viability.

Previous studies have shown that the substitution of key glutamic acid residues within the adenine-binding loop can reduce pADPr hydrolysis activity in cell-free assays, although it does not abolish it entirely. Replacing glutamic acid with asparagine in bovine PARG (E^728^N) reduced catalytic activity to 18%, while the corresponding E^720^N mutation in mouse PARG retained approximately 60% activity [13,25]. In human PARG, mutations of I^726^P and E^727^A in the same loop reduced the activity to approximately 20% and 7%, respectively [8]. These findings suggest that the loop plays a facultative role in orientating the terminal adenine of the (n) ADPr unit for productive binding. In contrast, our study demonstrates that the deletion of the entire loop leads to a complete loss of enzymatic activity, highlighting its indispensable role in pADPr recognition and catalysis. This loss of function is likely due to not only impaired positioning but also a disruption in specific molecular interactions. Structural modeling predicts that the TYEG^717^ deletion and following structural changes in adenine-binding TIEG^721^ loop will disrupt the orientation of the adenine base for π-stacking interactions with a conserved phenylalanine residue in the active site. The disruption of this alignment likely interferes with the catalytic turnover and release of reaction products. Moreover, our findings support the hypothesis that substrate selectivity, particularly for adenine-containing ADPr units, may be mediated through recognition by TIEG^721^ residues, consistent with a previously proposed model [10].

PARG exhibits two distinct glycohydrolase activities: exo-glycohydrolase, which binds and cleaves the terminal ADP-ribose unit from the distal end of the poly(ADP-ribose) (pADPr) chain, and endo-glycohydrolase, which binds internally within the chain and cleaves internal ADPr linkages. Endo-hydrolysis has been proposed to occur through a processive mechanism, where PARG remains bound to the pADPr chain, thereby accelerating the overall hydrolysis rate [8,25]. Although both activities have been demonstrated in higher eukaryotes, the biological predominance and regulation of each mode remain unclear. Some models suggest that PARG initiates degradation with endo-activity, subsequently switching to exo-cleavage, while others indicate limited endo-cleavage and a predominant exo-hydrolytic role [8,30]. Our computational modeling suggests that the PARG^29b^ variant harbors a TIEG^721^ sequence in place of the TYEG^717^ motif. This includes both the deletion of the internal ADPr binding TIEG^721^ loop and a tyrosine-to-isoleucine substitution in the outer TYEG^717^ loop. These alterations likely impair binding to both the terminal (n) and internal (n + 1) ADPr units, potentially disrupting both exo- and endo-glycohydrolase functions.

Previous studies have shown that mammalian and Drosophila PARG enzymes differ in their ability to hydrolyze terminal mADPr. While Drosophila PARG can remove the terminal ADPr unit, in mammals, this function is primarily carried out by other hydrolases [31]. In our experimental system, however, we did not observe striking phenotypic differences attributable to this divergence. Notably, mouse PARG fully compensated for Drosophila PARG under normal physiological conditions, successfully rescuing developmental progression and supporting a normal lifespan in transgenic flies. Future studies are needed to determine whether this reflects a lack of functional importance of mADPr in Drosophila or a requirement for its removal only under specific stress or developmental conditions or whether mouse PARG acquires the ability to hydrolyze terminal mADPr in vivo due to specific posttranslational modifications, interacting partners, or other context-dependent factors.

Our findings reveal a striking divergence between cellular viability and organismal development in the context of PARG inactivation. Although the *Parg^29b^* mutation leads to a complete loss of enzymatic activity, mutant ESCs maintained normal proliferation, cell cycle dynamics, and apoptotic profiles in vitro. In contrast, the disruption of PARG function by this mutation in *Drosophila melanogaster* resulted in complete developmental arrest, underscoring the enzyme’s essential role during organismal development despite its apparent dispensability for basic cellular maintenance.

In conclusion, our findings define a key structural determinant of PARG enzymatic activity and establish a genetically tractable model for dissecting pADPr turnover in vivo. By pinpointing a single conserved loop essential in catalysis, this work opens up new avenues for understanding how polymer length, structure, and enzyme–substrate dynamics influence poly(ADP-ribose) metabolism in both physiological and pathological contexts. Moreover, the *Parg^29b^* ESC line could serve as a valuable tool for future studies on chromatin regulation and PARP inhibitor sensitivity in the absence of effective pADPr degradation.

## Figures and Tables

**Table 1:** gRNAs targeting Parg exons.

Number	Sequence
1	GGCAAACGGATCCCACACAG
2	AGAATGGTGAGCGAACTGCA
3	GGAACAAAACTTGTACCCTG
4	TGCGATTCTGAAATACAATG
5	TCACTGTGGTCATCAGTGTG
6	TCCTTTAGTATCCATCCAAG
7	TCGGCACCAACATCTGACAA
8	GATTTATCAAGATTTAGCTG
9	CAAATGGAGGCGAATCACCT
10	TCAGGTACTTGAAGAAGCAG
11	TGTTTGAAGGACGTTCATCA
12	ACGCTTACACGTCACTTACG
13	TCATTCTGTCACGATGTCAC
14	AAGTGAGCCTGAGTCACCAA
15	GTAAATGTCACCAATCCTGT
16	TGGAGGTGGTGTGACTGGTG
17	TGTTTCACGGCTGTTCACTG
18	GAACAGTACAGTGAATACAC
19	CGATTGGCAGCGGCGCTGCA
20	TCTCAGGCACAAACTGATCG
21	TTTCAGAAGGAACTCCAGGA
22	ACCACAGCCCCAGTTTCCCG
23	TGCACACTTTCCTTACCGAG

## Data Availability

All data is available upon request.

## Supplementary Materials

The following supporting information can be downloaded at https://www.mdpi.com/article/10.3390/cells14141049/s1, Figure S1: Prediction of PARG binding pADPr; Figure S2: PARP inhibitors reduce the pADPr-related fluorescence in *Parg*-mutated ESCs; Figure S3: PARG^29b^ exhibits a similar level of expression and stability compared to wild-type version; Figure S4: Different ESC clones with *Parg^29b^* mutation exhibit pADPr accumulation in nuclei; Figure S5: Prediction of PARG^29b^ structure; Figure S6: *Parg^29b^* mutant ESCs retain the capacity to further elevate pADPr levels, indicating an excess of available free NAD^+^; Figure S7: Different versions of PARG exhibit a similar level of expression and stability compared to wild-type version; Figure S8: Uncropped versions of Western blotting.

## Abbreviations

The following abbreviations are used in this manuscript:

pADPr	Poly(ADP-ribose)
mADPr	Mono(ADP-ribose)
oADPr	Oligo(ADP-ribose)
PARP	Poly(ADP-ribose) Polymerase
PARG	Poly(ADP-ribose) Glycohydrolase
ESC(s)	Embryonic Stem Cell(s)
WT	Wild-Type
KO	Knockout
