# Supplementary material for: PARG Mutation Uncovers Critical Structural Determinant for Poly(ADP-Ribose) Hydrolysis and Chromatin Regulation in Embryonic Stem Cells
Source: Cells. 2025 Jul 9;14(14):1049. doi: 10.3390/cells14141049 (PMC12293112; doi:10.3390/cells14141049)
Supplement: Supplementary file 1 [file cells-14-01049-s001.zip › cells-3681298 supplyment update.pdf]

Supplementary Materials

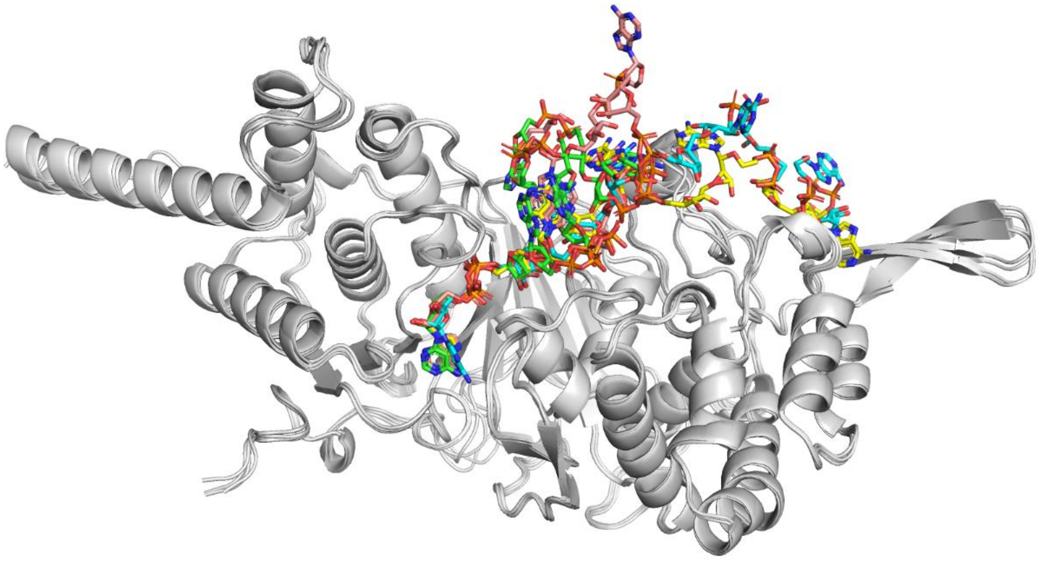

Figure S1. Prediction of PARG binding pADPr. Top four predictions of mouse PARG catalytic domain with five-unit oADPr by Chai. Catalytic domain is in gray, oADPr ligands from different predictions are in different colors.

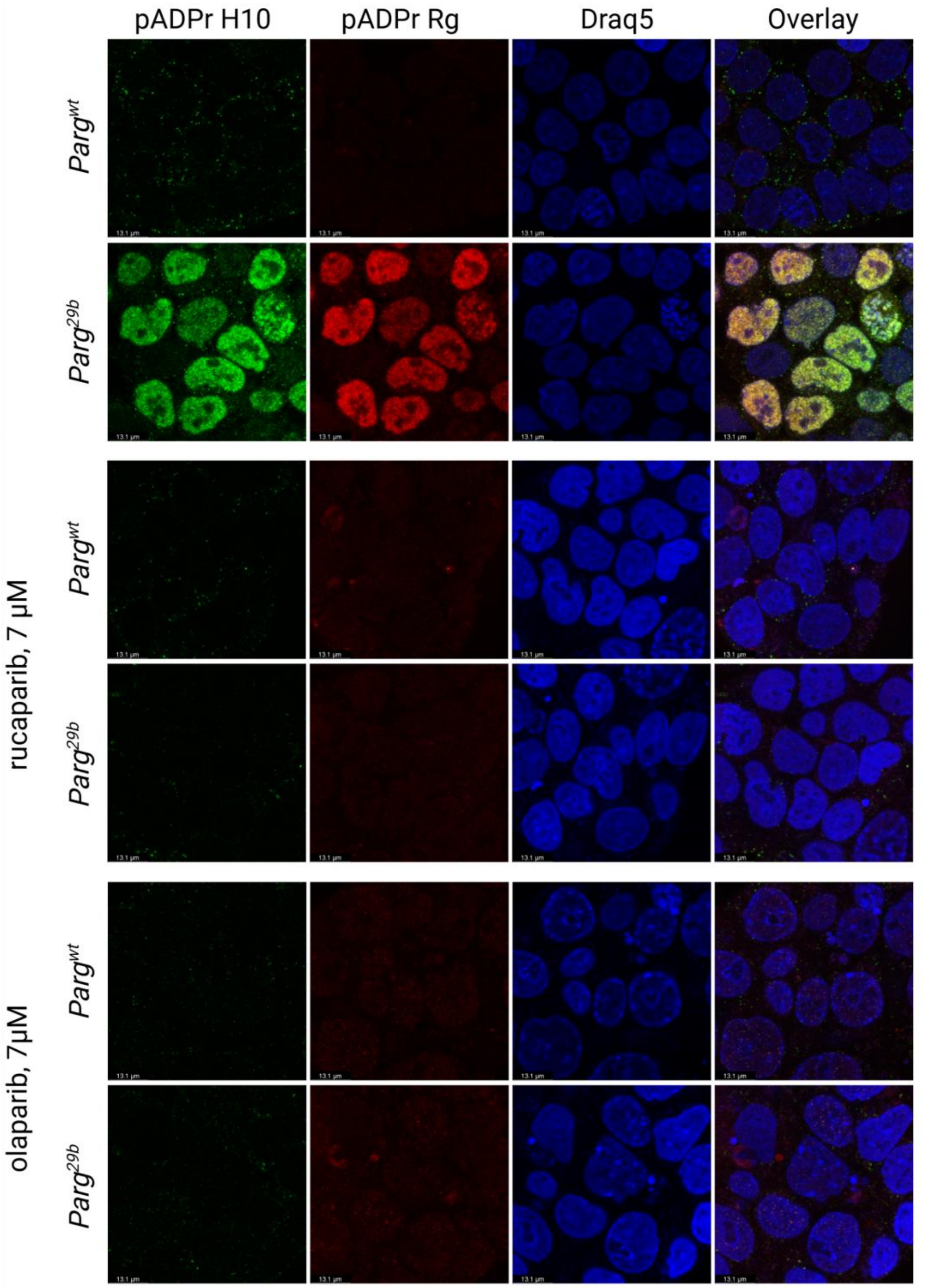

Figure S2. PARPs inhibitors reduces the pADPr-related fluorescence in *Parg* mutated ESCs cells. *Parg*^{29b} and control ESCs were grown with 7 µM of PARP inhibitors olaparib and rucaparib or with DMSO as vehicle for 24 hours. Cells were stained for pADPr with antibodies (H10) or special reagent (Rg). DNA was stained with Draq5.

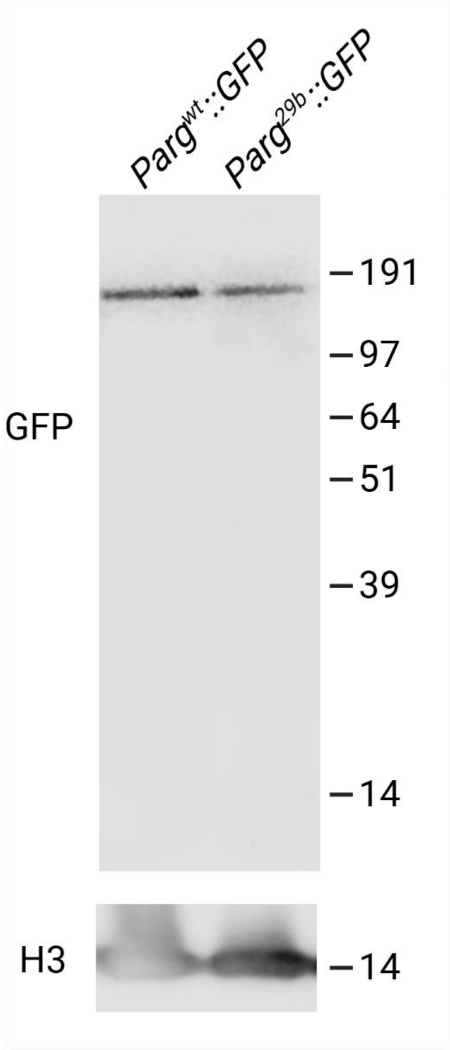

Figure S3. PARG^{29b} exhibits a similar level of expression and stability compared to wild type version. Western blotting of samples from *Parg^{wt}* and *Parg^{29b}* ESCs, in which the endogenous *Parg* gene was tagged with GFP with CRISPR/Cas9. Blots were probed with anti-GFP antibodies and anti-histone H3 as a loading control. No signs of degradation were observed for the PARG^{29b} protein, indicating comparable stability to the wild-type version.

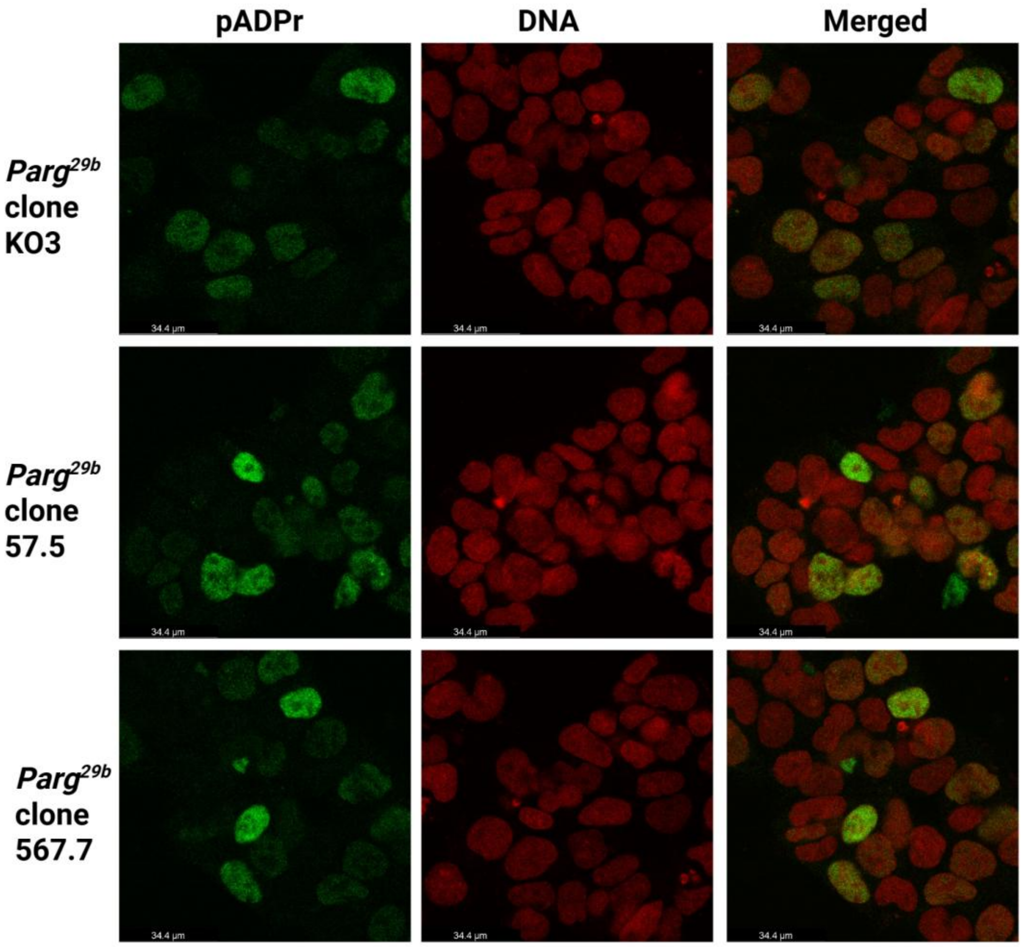

Figure S4. Different ESCs clones with *Parg*^{29b} mutation exhibit pADPr accumulation in nuclei. Staining of different Parg^{29b} clones of ESCs with antibodies against pADPr, demonstrating uneven pADPr levels between different nuclei. DNA marked with Draq5.

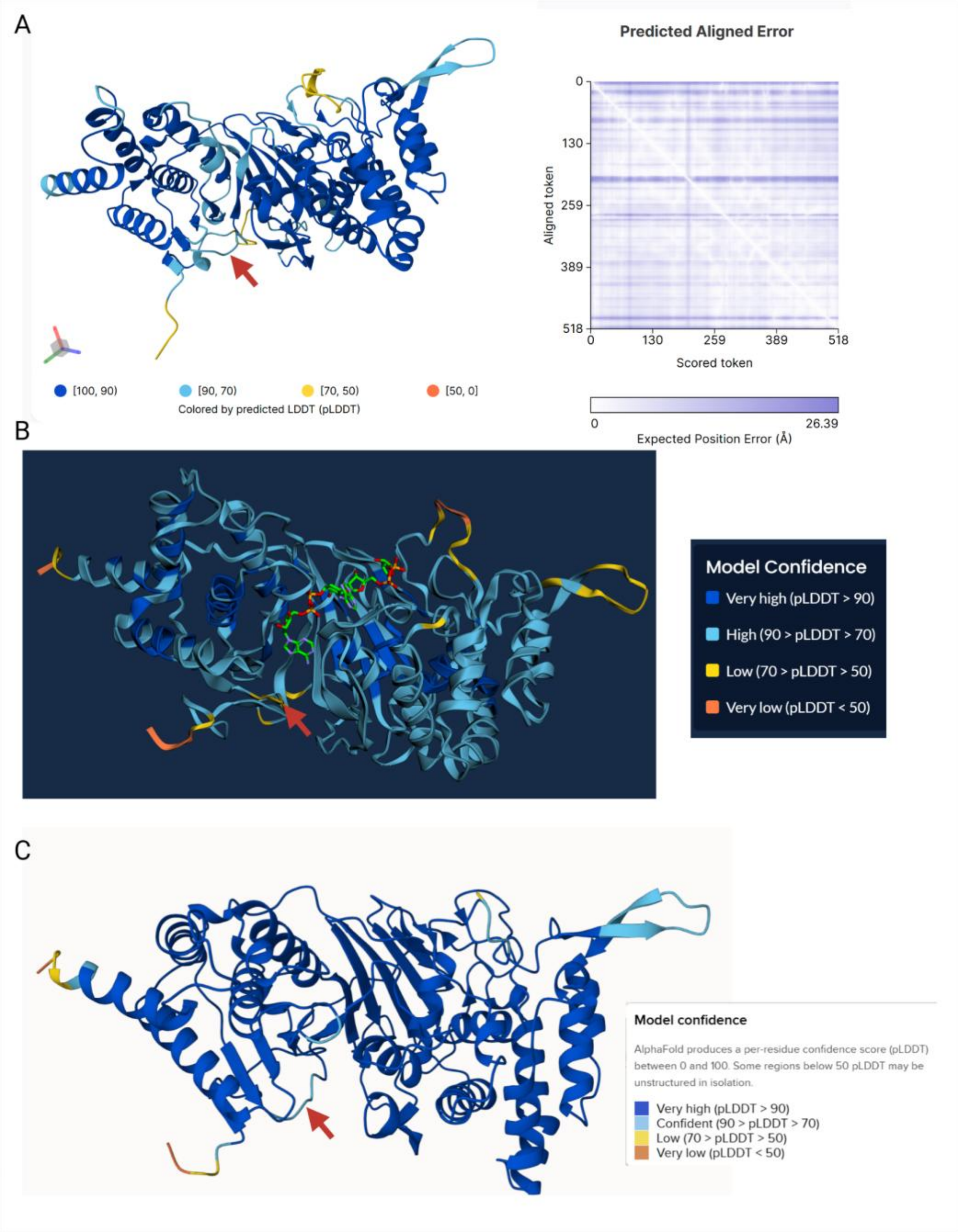

Figure S5. Prediction of PARG^{29b} structure. Structural models of PARG harboring the 29b deletion were generated using Chai (a), RosettaFold (b), and AlphaFold (c). All three prediction methods indicate that the overall fold of PARG remains largely unchanged. However, deletion of the TYEG^{717} loop in the 29b variant alters the positioning of the adjacent TIEG^{721} loop. The local confidence scores (pLDDT) for the affected region fall within the range of [90–70) for Chai and AlphaFold predictions, and [70–50) for RosettaFold. The region is highlighted with an arrow.

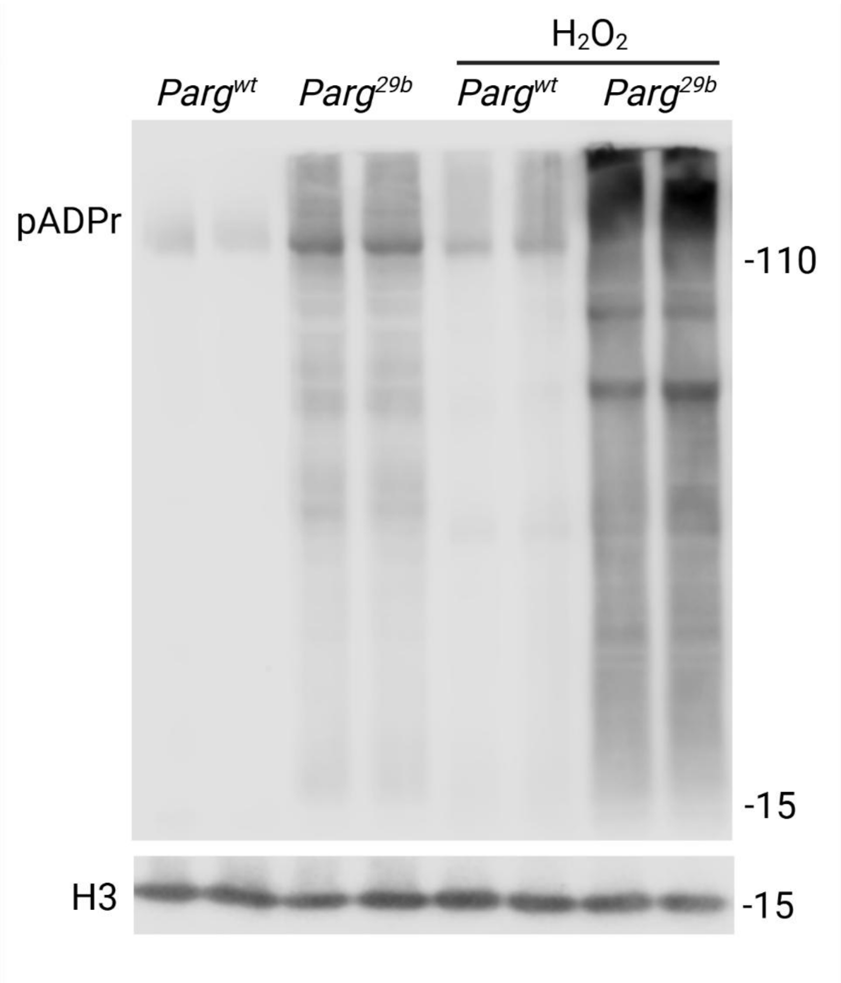

Figure S6. ***Parg**^{29b}* **mutant ESCs retain the capacity to further elevate pADPr levels, indicating an excess of available free NAD$^+$.** *Parg*^{wt} and *Parg*^{29b} ESCs were treated with 1mM hydrogen peroxide and samples were prepared 10 minutes after exposure. Western Blotting was performed, and samples were stained with special reagent against pADPr and against histone H3 as a loading control.

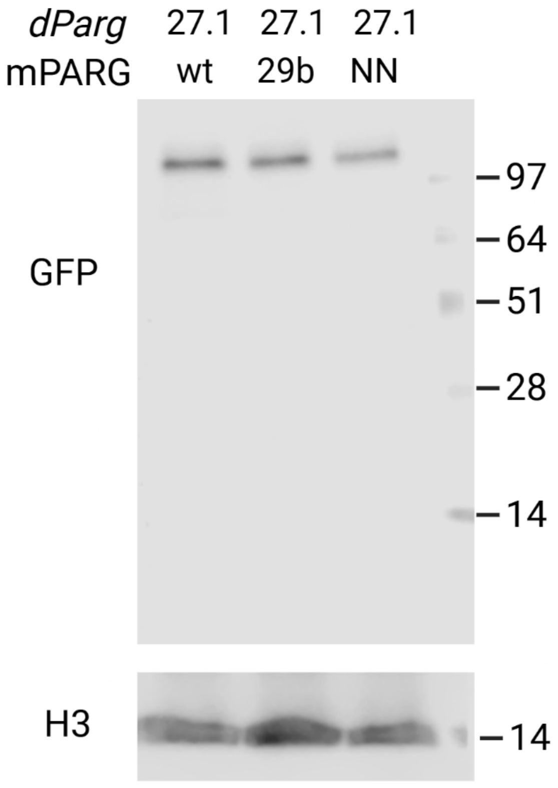

Figure S7 Different versions of PARG exhibit a similar level of expression and stability compared to wild type version. Western blotting of samples from transgenic flies carrying mouse $PARG^{wt}$, $PARG^{29b}$ or $PARG^{NN}$ tagged with GFP in a *Parg*$^{27.1}$ knockout background. Blots were probed with anti-GFP antibodies and anti-histone H3 as a loading control. No signs of degradation were observed for the $PARG^{29b}$ or $PARG^{NN}$ proteins, indicating comparable stability to the wild-type version.

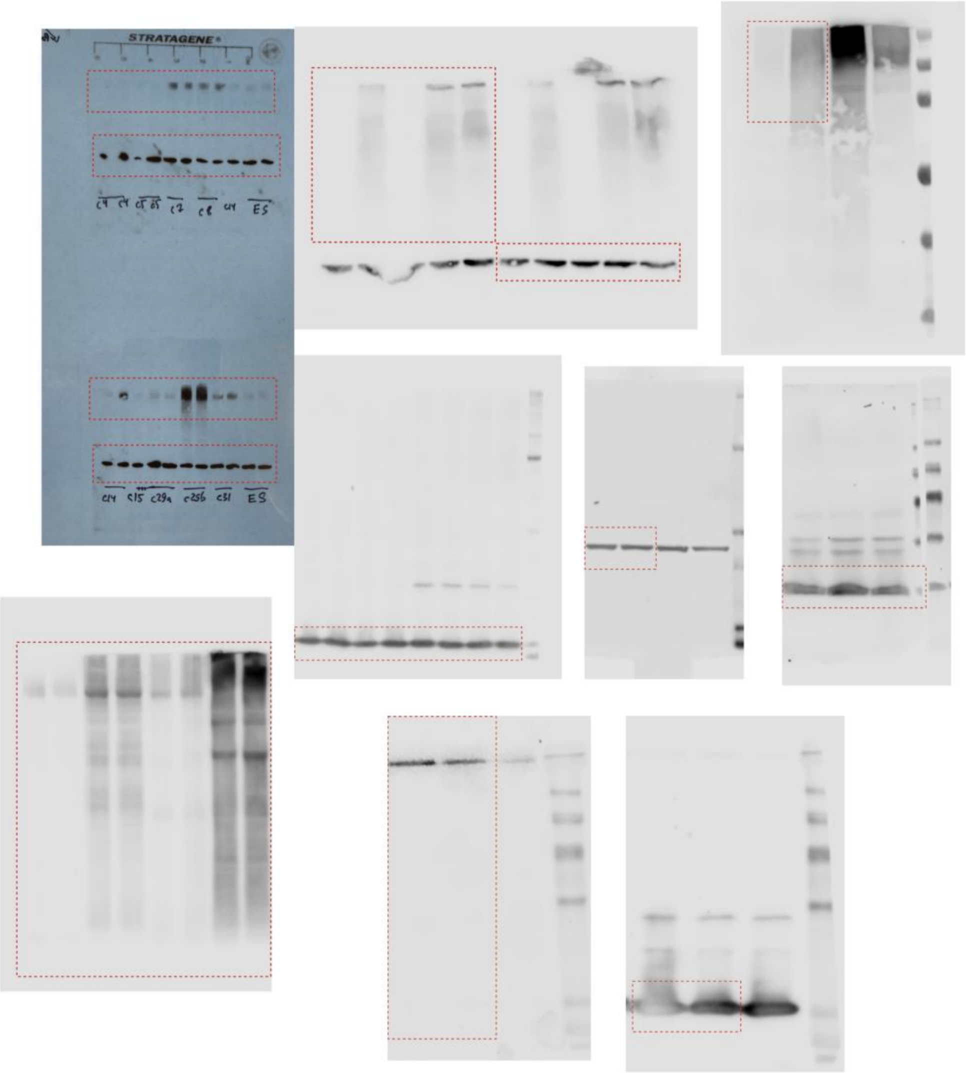

Figure S8. Uncropped versions of Western Blotting.
